# Impact of the Second Wave of the COVID-19 Pandemic on the Quality of Life and Emotional Well-being of Students studying humanities disciplines in Russia

**DOI:** 10.1192/j.eurpsy.2024.1065

**Published:** 2024-08-27

**Authors:** V. I. Rozhdestvenskiy, V. V. Titova, I. A. Gorkovaya, D. O. Ivanov, Y. S. Aleksandrovich

**Affiliations:** Department of Psychosomatics and Psychotherapy, Saint Petersburg State Pediatric Medical University, Saint Petersburg, Russian Federation

## Abstract

**Introduction:**

The second wave of the COVID-19 pandemic had a significant impact on the quality of life and emotional well-being of the Russian population, with increased emotional disorders such as depression and anxiety. This study focuses on the specific context of Russian university students studying humanities disciplines, who had to adapt to remote learning and self-education during the pandemic.

**Objectives:**

This study aimed to assess the quality of life and measure the levels of depression, anxiety, and stress among Russian humanities students. Additionally, it examined the correlations between quality of life and emotional disorders.

**Methods:**

Data collection was conducted between January and April 2021 using a customized Google form. The study included 35 students from Russian universities. Quality of life was assessed using the WHOQOL-BREF questionnaire, and levels of depression, anxiety, and stress were determined using the DASS-21 methodology, both adapted for use in Russia.

**Results:**

The mean values for the quality of life domains were as follows: “physical and psychological well-being” (M = 20.65±3.85), “self-image” (M = 19.21±3.54), “microsocial support” (M = 10.39±2.36), and “social well-being” (M = 27.93±4.15). Notably, 54% of respondents exhibited no symptoms of depression, 66% showed no signs of anxiety, and 69% reported no stress. Correlation analysis revealed that there was no statistically significant relationship between stress and quality of life, and social well-being did not correlate with emotional disturbances.

**Conclusions:**

During the second wave of the COVID-19 pandemic, the majority of Russian humanities students did not experience clinical manifestations of depression, anxiety, or stress. To improve their emotional well-being, students should prioritize their physical and psychological health, self-perception, self-esteem, and relationships with their immediate social circles, particularly their families. In this pandemic context, broader social factors such as recreational opportunities took a back seat in students’ priorities.

**Disclosure of Interest:**

None Declared

